# Functional Characterization of Odorant Binding Protein PyasOBP2 From the Jujube Bud Weevil, *Pachyrhinus yasumatsui* (Coleoptera: Curculionidae)

**DOI:** 10.3389/fphys.2022.900752

**Published:** 2022-04-27

**Authors:** Bo Hong, Qing Chang, Yingyan Zhai, Bowen Ren, Feng Zhang

**Affiliations:** ^1^ Bio-Agriculture Institute of Shaanxi, Xi’an, China; ^2^ Shaanxi Academy of Forestry, Xi’an, China

**Keywords:** *Pachyrhinus yasumatsui*, odorant binding protein, prokaryotic expression, host volatile, fluorescence competitive binding assay, molecular docking

## Abstract

Odorant binding proteins (OBPs) play an important role in insect olfaction. The jujube bud weevil *Pachyrhinus yasumatsui* (Coleoptera: Curculionidae) is a major pest of *Zizyphus jujuba* in northern China. In the present study, based on the antennal transcriptome, an OBP gene of *P. yasumatsui* (*PyasOBP2*) was cloned by reverse transcription PCR (RT-PCR). Expression profile analyses by quantitative real-time PCR (qRT-PCR) revealed that *PyasOBP2* was highly expressed in the antennae of both male and female *P. yasumatsui* adults, while its expression was negligible in other tissues. PyasOBP2 was prokaryotically expressed, and purified by Ni-NTA resin. The fluorescence competitive binding assays with 38 plant volatiles from *Z. jujuba* showed that PyasOBP2 could bind with a broad range of plant volatiles, and had strongest binding capacities to host-plant volatiles like ethyl butyrate (K_i_ = 3.02 μM), 2-methyl-1-phenylpropene (K_i_ = 4.61 μM) and dipentene (K_i_ = 5.99 μM). The three dimensional structure of PyasOBP2 was predicted by homology modeling, and the crystal structure of AgamOBP1 (PDB ID: 2erb) was used as a template. The molecular docking results indicated that the amino acid residue Phe114 of PyasOBP2 could form hydrogen bonds or hydrophobic interactions with some specific ligands, so this residue might play a key role in perception of host plant volatiles. Our results provide a basis for further investigation of potential functions of PyasOBP2, and development of efficient monitoring and integrated pest management strategies of *P. yasumatsui*.

## Introduction

In long-term interactions with external environments, insects have evolved a highly specific and sensitive olfactory system, which enables them to sense various chemical signals and undertake a series of behaviors such as mating, host location, foraging, oviposition, and predator avoidance (Justice et al., 2010; Elgar et al., 2018). The olfactory system consists of various proteins expressed during the chemoreceptive process, such as odorant binding proteins (OBPs), chemosensory proteins (CSPs), olfactory receptors (ORs), gustatory receptors (GRs), ionotropic receptor (IRs), sensory neuron membrane proteins (SNMPs), and odorant degrading enzymes (ODEs) (Vosshall et al., 1999; De Bruyne and Baker, 2008; Sanchez-Gracia et al., 2009). OBPs and CSPs are both soluble proteins that are concentrated in the chemosensilla lymph of insects. The two kinds of proteins are able to selectively bind, and transport hydrophobic odorant molecules across the lymph to ORs located on the dendritic membrane of sensory neurons, activating the chemical signal transduction process (Laughlin et al., 2008; Leal, 2013; Pelosi et al., 2014).

In general, insect OBPs are small (about 100–200 amino acids) hydrosoluble proteins. According to distinct conserved cysteine patterns, insect OBPs can be divided into four subfamilies: “classic OBPs” with six conserved cysteine residues, “minus-C OBPs” with four conserved cysteine residues, “plus-C OBPs” with eight conserved cysteine residues, and “atypical OBPs” with six conserved cysteine residues as in “classic OBPs”, but with additional cysteines in the C-terminal region (Hekmat-Scafe et al., 2002; Venthur et al., 2014; Brito et al., 2016).

Since the first insect OBP was identified from *Antheraea polyphemus* (Vogt and Riddiford, 1981), a large number of OBPs have been identified by using sequenced genomes and transcriptomes from several insect orders, including Diptera, Hymenoptera, Lepidoptera, Hemiptera, Coleoptera, and Orthoptera (Jacquin-Joly et al., 2000; Northey et al., 2016; Wu et al., 2016; Pelosi et al., 2018; Venthur and Zhou, 2018). In recent years, an increasing number of studies involving the identification and function of OBP genes in insect species have demonstrated that most insect OBPs are highly expressed in antennae, indicating that OBPs play a key role in chemoreception (Wang et al., 2019; Zhang et al., 2020). Moreover, OBPs are found to selectively bind to various volatiles emitted from host plants (Deng et al., 2012; Ju et al., 2012; Cui et al., 2018). Therefore, host volatiles play a crucial role in insect orientation and host selection, and studies on the binding characteristics of insect OBPs with volatiles will bring a better understanding of olfactory recognition mechanism at molecular levels.

The jujube bud weevil, *Pachyrhinus yasumatsui* (Kôno and Morimoto, 1960) (Coleoptera: Curculionidae), has recently become a major pest of jujube plants (*Ziziphus jujuba* Mill) in northern China, causing serious ecological damage and large economic losses (Huang and Li, 1993; Ren and Qi, 2009; Tang et al., 2013). Although being still the main tools to control *P. yasumatsui*, chemical insecticides pose serious threat to environmental and human health, and lead to pest resistance (Yang et al., 2019; Yan et al., 2020). The jujube bud weevil is an oligophagous herbivore, feeding mainly on jujube plants, so the host selection behaviors of this insect may rely on olfaction (Hong et al., 2017; Wang et al., 2017). In previous studies, *P. yasumatsui* adults were found to be significantly attracted by several volatiles emitted from jujube shoots (e.g., ocimene, α-farnesene, nonanal and methyl palmitate), based on electroantennography (EAG) and Y-tube olfactometer experiments (Yan et al., 2017; Yan et al., 2020). However, little is known about olfaction in this pest at the molecular level.

In our previous studies, 24 putative OBPs were identified from the antennal transcriptome of *Pachyrhinus yasumatsui* (unpublished), and the level of unigenes coding for OBPs was calculated using fragments per kilobase of transcript per million mapped read (FPKM) values. The FPKM values indicated that the candidate OBP gene *PyasOBP2* had the highest level in the male antennae (FPKM = 40599.96), suggesting that *PyasOBP2* was an antenna-enriched OBP gene and may be involved in the odor identification for *P. yasumatsui*. In this work, we cloned *PyasOBP2* by using RT-PCR, determined its expression profile in different tissues, and purified the recombinant protein to test its affinity with jujube volatiles by fluorescence competitive binding assays. Based on the results of ligand-binding assays, we performed three dimensional (3D) structural modeling and molecular docking to investigate the binding sites of PyasOBP2, and identify the key amino acid residues involved. Our results provide a foundation for clarifying molecular mechanisms of insect olfaction, and will serve as a reference for developing management strategies for this pest.

## Materials and Methods

### Experimental Insect Samples

The pupae of *P. yasumatsui* were collected from Jiaxian County, Shaanxi, China (37^°^59^′^53^″^N, 110^°^21^′^07^″^E) in April 2020, and placed in incubators at 25 ± 1°C, 16 h light: 8 h dark cycle and 60 ± 5% RH. The emerged male and female adults were collected and separately reared on fresh buds of *Zizyphus jujuba*. Antennae, heads (without antennae), thoraxes, abdomens, legs and wings of *P. yasumatsui* were collected from 5-d-old adults, immediately transferred to Eppendorf tubes immersed in liquid nitrogen, and stored at −80°C until RNA extraction.

### RNA Extraction, cDNA Synthesis, and Gene Cloning

Total RNA was extracted with the Trizol reagent (TaKaRa Co., Dalian, China). The integrity of RNA was assessed by 1.0% agarose gel electrophoresis, and the concentration was quantified with a SimpliNano spectrophotometer (GE Healthcare, Piscataway, NJ, United States). cDNA was synthesized from total RNA (1 μg for each sample) using the PrimeScript™ 1st Strand cDNA Synthesis Kit (TaKaRa) by following the manufacturer’s instructions, and cDNA samples were stored at −20°C.

The *OBP2* gene sequence was obtained from the antennal transcriptome of *P. yasumatsui* (GenBank No. SRR7871392, unpublished), and specific PCR primers were designed to amplify the coding region (Table 1). RT-PCR reactions were carried out by using the following conditions: 3 min at 95°C; 35 cycles of 30 s at 95°C, 30 s at 56°C, 30 s at 72°C; and 72°C for 10 min. The purified RT-PCR product was ligated into the pMD^®^19-T vector, and transformed into DH5α competent cells (TaKaRa) for sequencing.

**TABLE 1:** Primer pairs used for cloning, prokaryotic expression and gene expression analyses.

Primer name	Primer sequence (5′-3′)	Product size (bp)
**For gene cloning**		
OBP2-F	ATA​TTT​TGA​TTG​ACA​ATC​TAG​TCA​GAC	588
OBP2-R	ACT​TAG​ATT​TGG​GAT​GCG​TAT​T	
**For qRT-PCR**		
OBP2-qF	GTG​GAA​TCA​CGG​AGG​ACG​A	161
OBP2-qR	ATC​TTT​GAA​TGG​ATA​CGG​TTG​TG	
EF1α-qF	TCC​CAA​GCT​GAT​TGT​GCT​G	112
EF1α-qR	CAA​GGG​TGA​AGG​CGA​GAA​G	
Actin-qF	TGTTGCGGCTCTTGTCGT	169
Actin-qR	GCT​TTG​GGC​TTC​ATC​TCC​TA	
**For prokaryotic expression**		
OBP2-eF	CGGGA​TCCAAG​CTT​ACA​TTG​CCA​CCA​GAA​T	351
OBP2-eR	CCGGAA​TTCCGG​TTA​GAC​GAA​GAA​CCA​ATT​CTC​AGG	

Restriction sites are underlined.

### Sequence and Phylogenetic Analyses

The open reading frame (ORF) of *PyasOBP2* was predicted by using the ORFfinder (https://www.ncbi.nlm.nih.gov/orffinder/). The signal peptide of the amino acid sequence of PyasOBP2 was predicted using the SignalP program server (https://services.healthtech.dtu.dk/service.php?SignalP-5.0). The molecular weight and theoretical isoelectric point of PyasOBP2 was calculated with the Expasy program online (http://web.expasy.org/protparam/). Sequence alignment of PyasOBP2 with OBPs from other insects was carried out with DNAMAN 9.0 (Lynnon Biosoft, San Ramon, CA, United States). Based on amino acid sequences of PyasOBP2 and other coleopteran OBPs, phylogenetic analyses were performed in MEGA X (Kumar et al., 2018) using the neighbor-joining approach with a bootstrap replication of 1000. Finally, the phylogenetic tree was created and edited with FigTree 1.4.4 (http://tree.bio.ed.ac.uk/software/figtree/).

### Tissue Expression of *PyasOBP2*

Tissue expression levels of *PyasOBP2* in *P. yasumatsui* were measured by qRT-PCR. The *EF1-α* gene (GenBank No. OK105108) and *β-actin* gene (GenBank No. OK322363) from *P. yasumatsui* were used as reference genes. Primer sequences were designed with Primer-BLAST (https://www.ncbi.nlm.nih.gov/tools/primer-blast/), and listed in Table 1. qRT-PCR reactions were performed with TB Green^®^ Premix Ex Taq^TM^ II (TaKaRa) and a StepOnePlus Real-Time PCR System (Applied Biosystems, Carlsbad, CA, United States) using the following conditions: 30 s at 95°C, followed by 40 cycles of 5 s at 95°C, 30 s at 60°C, 30 s at 72°C. Three biological replicates and three technical replicates were conducted for each gene. The expression level (*L*) of all the genes was calculated with Eq. 1. *L =* the expression level of all the genes, *Ct* = the threshold cycle value, *E* = amplification efficiency. The normalized expression level of *PyasOBP2* (*N*_*OBP2*_) in different adult tissues was calculated with Eq. 2 (Livak and Schmittgen, 2001; Vandesompele et al., 2002). Significant differences in different tissues were analyzed by one-way ANOVA, followed by the Tukey’s HSD tests (*p* < 0.05 was considered statistically significant). The Student’s *t*-test was used to compare the expressions of *PyasOBP2* between male and female adults. All the data were analyzed using the SPSS 22.0 software.

$$L={\left(1+E\right)}^{-Ct}$$ (1)

$$N_{OBP2}=\frac{{\left(1+E_{OBP2}\right)}^{-Ct_{OBP2}}}{\sqrt{{\left(1+E_{EF-1\alpha }\right)}^{-Ct_{EF-1\alpha }}\times {\left(1+E_{\beta -actin}\right)}^{-Ct_{\beta -actin}}}}$$ (2)

### Cloning and Construction of Recombinant Plasmids

Primers with restriction enzyme sites *BamH*I and *EcoR*I were designed with Primer Premier 5.0 (Table 1), and the coding region of PyasOBP2 without the signal peptide was amplified with PCR. The PCR products were ligated into the pMD^®^19-T vector, transformed into DH5α competent cells (TaKaRa Co., Dalian, China) and then sequenced. The correct pMD^®^19-T plasmids were digested by restriction enzymes (*BamH*I and *EcoR*I) (TaKaRa) for 1–2 h at 37°C, cloned into the digested pET32a vector, and then transformed into DH5α cells. The correct recombinant plasmids were transformed into BL21 (DE3) competent cells (TaKaRa). Single colonies were cultured in liquid LB (supplemented with 100 mg/ml ampicillin) overnight at 37°C.

### Prokaryotic Expression and Purification of PyasOBP2

The culture was diluted 1:100 with liquid LB, and incubated at 37°C until the OD_600_ reached a value of 0.6–0.8. Protein expression was induced by adding isopropyl-β-D-1-thiogalactopyranoside (IPTG) at a final concentration of 0.5 mM into the culture, and allowed to last for 10 h at 18°C. The bacterial cells (500 ml) were collected by centrifugation (8000 g for 10 min, 4°C), and the cell pellet was suspended with the lysis buffer (50 mg/ml Lysozyme and 20 mM Tris-HCl buffer at pH 7.4). The suspension was sonicated on ice, and centrifuged (12000 g for 30 min, 4°C) for a second time. Recombinant PyasOBP2 was examined by Sodium Dodecyl Sulfate—Polyacrylamide Gel electrophoresis (SDS-PAGE). Protein present in the supernatant was purified with a Ni-NTA His·Bind Resin column (7 Sea Biotech, Shanghai, China). The purified protein was assessed by SDS-PAGE, identified with the anti-His tag monoclonal antibody (Cwbio biotech, Beijing, China) by the Western Blot analysis, and desalted in a dialysis buffer (20 mM Tris-HCl at pH 7.4). To avoid confounding effects on subsequent experiments, His-tag was removed from the protein using a recombinant enterokinase (rEK) (Yeasen Biotech, Shanghai, China), and the concentration of the protein was assayed by the BCA protein quantification kit (Cwbio biotech, Beijing, China).

### Fluorescence Binding Assays

Fluorescence competitive binding assays were carried out on an F-2700 fluorescence spectrophotometer (Hitachi, Tokyo, Japan) to determine the binding affinity of PyasOBP2. 1-N-phenyl-naphthylamine (1-NPN) was used as a fluorescent probe (Pelosi et al., 2006) with excitation at 337 nm, and the emission spectra were measured from 370 to 550 nm. Based on gas chromatography-mass spectrometry (GC-MS) and previous studies (Yan et al., 2017; Yan et al., 2020), 38 volatiles derived from *Z. jujuba* were selected as putative ligands for fluorescence competitive binding assays (Table 2). 1-NPN and all ligands used in binding assays were diluted in methanol to 1 mM stock solutions. To measure the binding affinity of PyasOBP2 with 1-NPN, PyasOBP2 solution (with a final concentration of 2 μM) was titrated with 1-NPN to final concentrations ranging from 2 to 20 μM. In ligand-binding assays, each ligand with a concentration ranging from 0 to 20 μM was added into a mixture of PyasOBP2 (2 μM) and 1-NPN (2 μM), and maximal fluorescence intensities were plotted against ligand concentrations based on three replicates.

**TABLE 2:** Binding affinities of PyasOBP2 to jujube volatile ligands in fluorescence binding assays.

Ligands	Formula	CAS	Source/Purity	IC_50_(μM)	K_i_(μM)
**Alcohols**					
1-Penten-3-ol	C_5_H_10_O	616-25-1	Aladdin, >97.0%	>20	-
*cis*-3-Hexen-1-ol	C_6_H_12_O	928-96-1	Aladdin, 98.0%	8.06	6.85
*trans*-2-Hexen-1-ol	C_6_H_12_O	928-95-0	Aladdin, 97.0%	8.93	6.60
Benzyl alcohol	C_7_H_8_O	100-51-6	Aladdin, ≥99.5%	16.18	11.96
Eucalyptol	C_10_H_18_O	470-82-6	Aladdin, >99.5%	15.07	12.81
Linalool	C_10_H_18_O	78-70-6	Aladdin, 98.0%	11.62	9.88
Nerolidol	C_15_H_26_O	7212-44-4	Aladdin, 97.0%	7.54	6.41
**Terpenoids**					
Ocimene	C_10_H_16_	13877-91-3	Sigma, ≥90.0%	10.49	8.92
α-Pinene	C_10_H_16_	7785-26-4	Aladdin, ≥99.0%	10.80	9.18
Camphene	C_10_H_16_	79-92-5	Aladdin, 95.0%	8.33	7.08
α-Phellandrene	C_10_H_16_	99-83-2	Sigma, >95.0%	10.73	9.12
Myrcene	C_10_H_16_	123-35-3	Aladdin, ≥90.0%	11.80	8.92
Dipentene	C_10_H_16_	7705-14-8	Aladdin, 95.0%	7.05	5.99
3-Carene	C_10_H_16_	13466-78-9	Aladdin, >90.0%	>20	-
β-Caryophyllene	C_15_H_24_	87-44-5	Sigma, ≥98.0%	>20	-
Squalene	C_30_H_50_	111-02-4	Aladdin, 98.0%	>20	-
**Esters**					
Ethyl butyrate	C_6_H_12_O_2_	105-54-4	Aladdin, ≥99.5%	3.55	3.02
Butyl acetate	C_6_H_12_O_2_	123-86-4	Aladdin, ≥99.7%	>20	-
Ethyl 2-methylbutyrate	C_7_H_14_O_2_	7452-79-1	Aladdin, 98.0%	>20	-
Ethyl valerate	C_7_H_14_O_2_	539-82-2	Aladdin, ≥99.7%	>20	-
Ethyl isovalerate	C_7_H_14_O_2_	108-64-5	Aladdin, ≥99.7%	>20	-
*cis*-3-Hexenyl acetate	C_8_H_14_O_2_	3681-71-8	Aladdin, 98.0%	>20	-
*cis*-3-Hexenyl 3-methylbutanoate	C_11_H_20_O_2_	35154-45-1	Aladdin, 97.0%	>20	-
2-Methylbutyric Acid *cis*-3-Hexen-1-yl Ester	C_11_H_20_O_2_	53398-85-9	Aladdin, 98.0%	11.92	10.13
Dibutyl phthalate	C_16_H_22_O_2_	84-74-2	Aladdin, >99.5%	10.46	7.73
Methyl palmitate	C_17_H_34_O_2_	112-39-0	Aladdin, ≥99.0%	>20	-
Methyl oleate	C_19_H_36_O_2_	112-62-9	Aladdin, ≥99.0%	>20	-
**Aldehydes**					
Isobutyraldehyde	C_4_H_8_O	78-84-2	Aladdin, >99.5%	12.99	11.04
*trans*-2-Hexen-1-al	C_6_H_10_O	6728-26-3	Aladdin, 98.0%	15.75	13.39
Caproaldehyde	C_6_H_12_O	66-25-1	Aladdin, ≥99.0%	10.86	8.03
Heptaldehyde	C_7_H_14_O	111-71-7	Aladdin, ≥98.0%	15.37	11.36
Octanal	C_8_H_16_O	124-13-0	Aladdin, 99.0%	7.81	6.64
Nonanal	C_9_H_18_O	124-19-6	Aladdin, 96.0%	15.56	13.22
**Others**					
Dodecane	C_12_H_26_	112-40-3	Aladdin, ≥99.5%	>20	-
2-Methyl-1-phenylpropene	C_10_H_12_	768-49-0	Aladdin, >98.0%	5.42	4.61
Benzonitrile	C_7_H_5_N	100-47-0	Aladdin, ≥99.5%	>20	-
Geranyl nitrile	C_10_H_15_N	5146-66-7	Aladdin, 97.0%	>20	-
Eugenol	C_10_H_12_O_2_	97-53-0	Aladdin, >99.5%	>20	-

The dissociation constant K_1-NPN_ (for PyasOBP2 binding with 1-NPN) was calculated with Scatchard plotting of binding data in the GraphPad Prism 8.0 Software (Sideris et al., 1992). The dissociation constant (K_i_) of each ligand was calculated with Eq. 3, as described by Cui et al. (2018). The ligand binding affinity to PyasOBP2 was considered as very strong (K_i_≤5 μM), strong (5 μM<K_i_≤10 μM), moderate (10 μM<K_i_≤20 μM) and weak (K_i_>20 μM) in this study.

$$K_{i}=IC_{50}/\left(1+\left[1-\mathrm{NPN}\right]/K_{1-\mathrm{NPN}}\right)$$ (3)

### Three Dimensional Structural Modeling and Molecular Docking

Structural templates for PyasOBP2 were searched by using PSI-BLAST against the Protein Data Bank (PDB) database. Based on high sequence similarity with PyasOBP2, the crystal structure of AgamOBP1 from *Anopheles gambiae* (PDB ID: 2erb) was selected as a template for homology modeling using Modeller 10.1 (Webb and Sali, 2016). To obtain the reliable 3D structure of PyasOBP2, the quality of models was assessed by Verify3D and PROCHECK (https://saves.mbi.ucla.edu/).

The 3D models of selected ligands were generated and optimized using ChemBioDraw12.0 (Cousins, 2011). The molecular docking of PyasOBP2 with ligands was performed using Autodock 4.2.6 (https://autodock.scripps.edu/). LigPlot + v.2.2.4 (https://www.ebi.ac.uk/thornton-srv/software/LigPlus/) and PyMOL v.2.5.2 (https://pymol.org/) were used to visualize 2D and 3D structures of the proteins, respectively.

## Results

### Characterization of *PyasOBP2* cDNA

The full-length cDNA of *PyasOBP2* (GenBank No. OK322360) was obtained by RT-PCR using specific primers. The cDNA sequence of *PyasOBP2* contained a 408-bp ORF encoding 135 amino acid residues. At the N-terminus, PyasOBP2 possessed a predicted 19-residue signal peptide (Supplementary Figure S1). The predicted molecular weight and theoretical isoelectric point (pI) of the mature protein PyasOBP2 were 13.71 kDa and 4.98, respectively.

**FIGURE 1:**
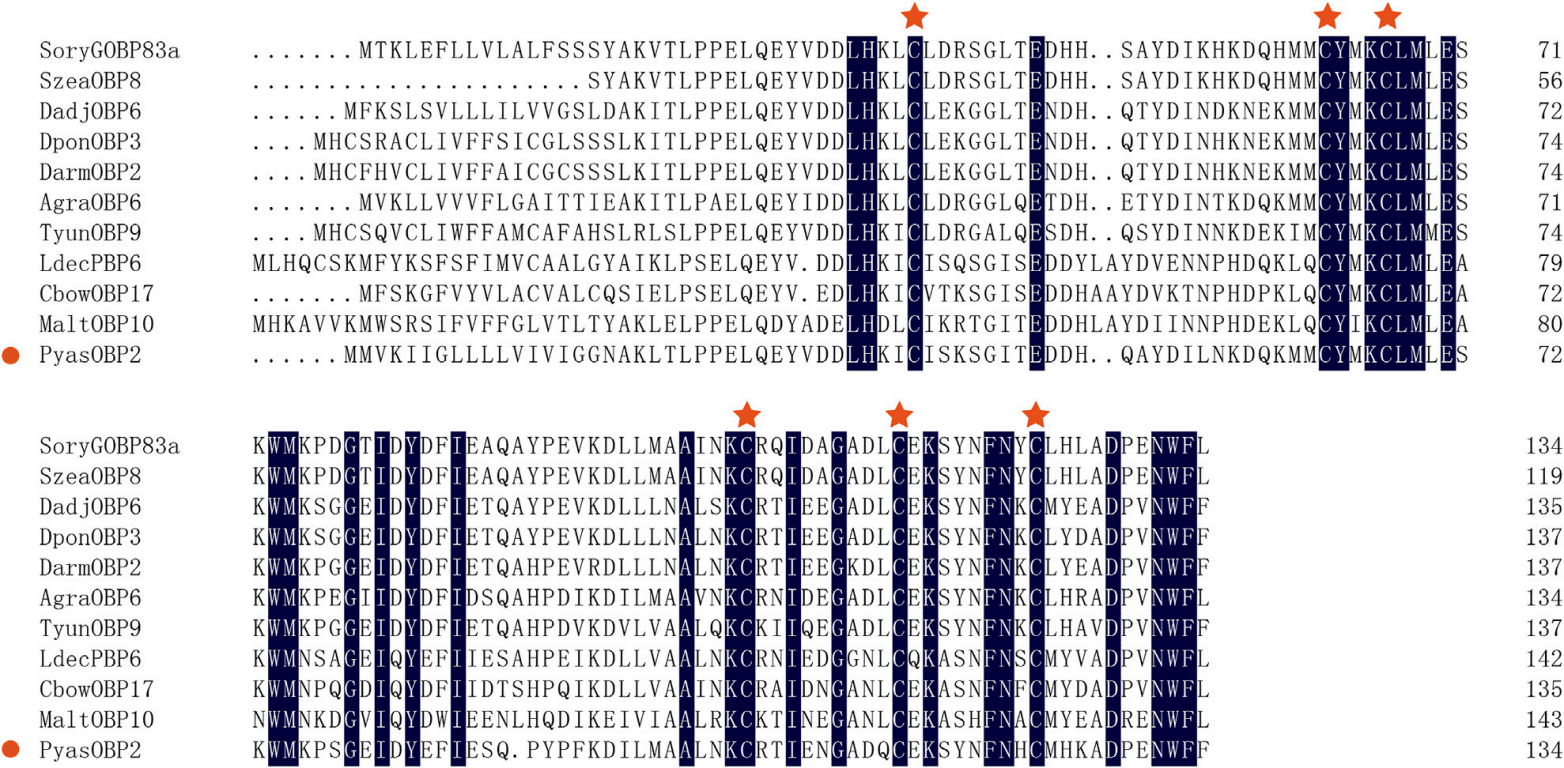
Multiple alignments of PyasOBP2 and other OBPs from coleopterans. The conserved cysteine residues were marked with a red star. Species names and GenBank accession numbers of the ten OBPs are: *Sitophilus oryzae* (SoryGOBP83a, XP_030747957); *Sitophilus zeamais* (SzeaOBP8, QCT83262); *Dendroctonus adjunctus* (DadjOBP6, QKV34987); *Dendroctonus ponderosae* (DponOBP3, AKK25131); *Dendroctonus armandi* (DarmOBP2, AIY61045); *Anthonomus grandis* (AgraOBP6, AVI04887); *Tomicus yunnanensis* (TyunOBP9, AMP19491); *Leptinotarsa decemlineata* (LdecPBP6, XP_023024287); *Colaphellus bowringi* (CbowOBP17, ALR72505); *Monochamus alternatus* (MaltOBP10, AIX97025).

**FIGURE 2:**
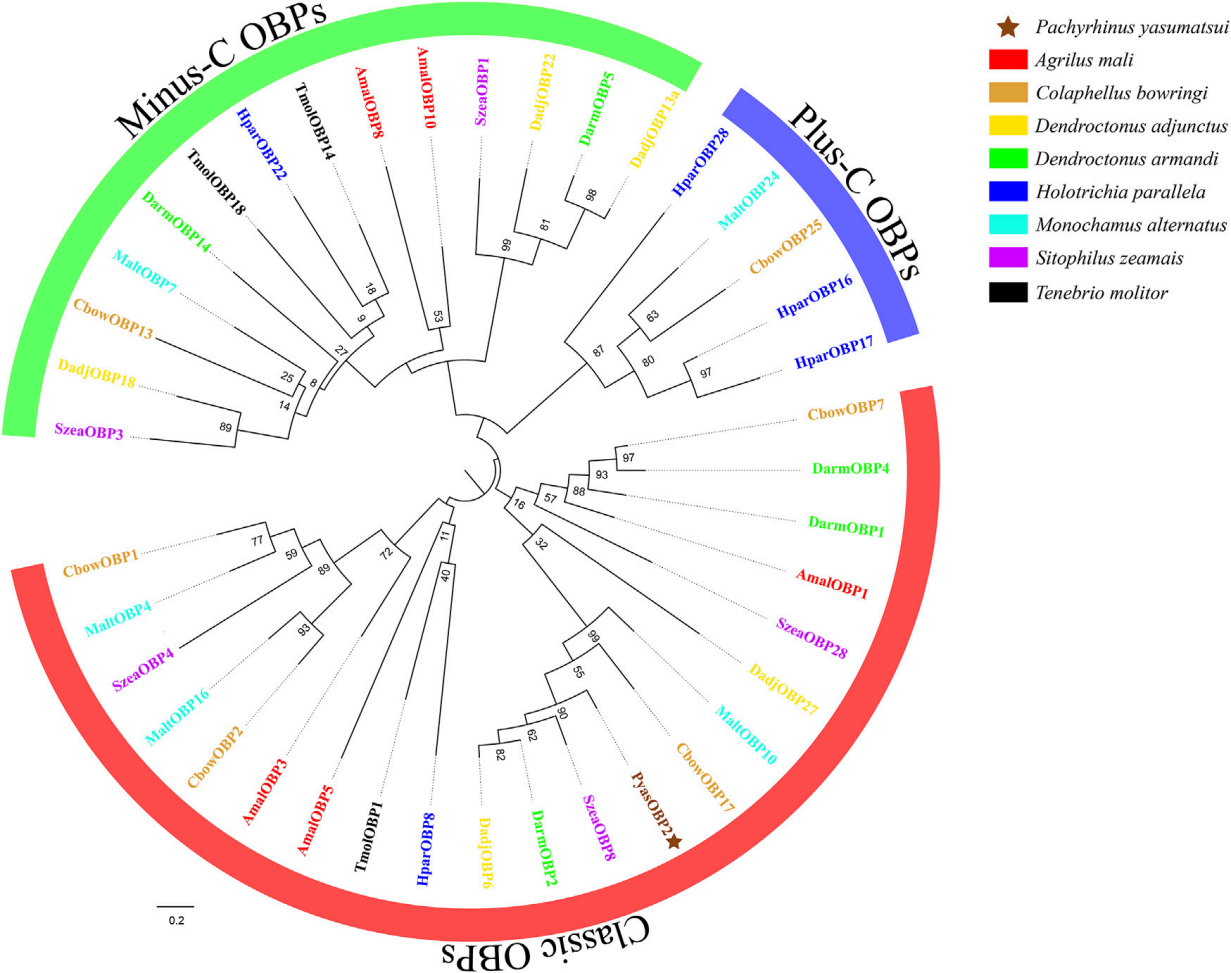
Phylogenetic tree of PyasOBP2 and OBPs from other coleopterans. Gene names and GenBank accession numbers of 40 OBPs are as follows: *Pachyrhinus yasumatsui* (PyasOBP2, MG585343); *Agrilus mali* (AmalOBP1, AVU05010; AmalOBP3, AVU05012; AmalOBP5, AVU05014; AmalOBP8, AVU05017; AmalOBP10, AVU05019); *C. bowringi* (CbowOBP1, ALR72489; CbowOBP2, ALR72490; CbowOBP7, ALR72495; CbowOBP13, ALR72501; CbowOBP17, ALR72505; CbowOBP25, ALR72513); *D. adjunctus* (DadjOBP6, QKV34987; DadjOBP13a, QKV34993; DadjOBP18, QKV34997; DadjOBP22, QKV34999; DadjOBP27, QKV35002); *D. armandi* (DarmOBP1, AIY61044; DarmOBP2, AIY61045; DarmOBP4, ALM64966; DarmOBP5, ALM64967; DarmOBP14, ALM64972); *Holotrichia parallela* (HparOBP8, AKI84366; HparOBP16, AKI84374; HparOBP17, AKI84375; HparOBP22, AKI84380; HparOBP28, ALP75941); *M. alternatus* (MaltOBP4, AHA39269; MaltOBP7, AIX97022; MaltOBP10, AIX97025; MaltOBP16, AIX97031; MaltOBP24, AIX97039); *S. zeamais* (SzeaOBP1, QCT83255; SzeaOBP3, QCT83257; SzeaOBP4, QCT83258; SzeaOBP8, QCT83262; SzeaOBP28, QCT83282); *Tenebrio molitor* (TmolOBP1, AJM71475; TmolOBP14, AJM71488; TmolOBP18, AJM71492)

Sequence alignments of PyasOBP2 with ten homologous OBPs from other coleopteran insects revealed that PyasOBP2 had typical characteristics of classic OBPs with six conserved cysteine residues (C_1_-X_24_-C_2_-X_3_-C_3_-X_36_-C_4_-X_9_-C_5_-X_8_-C_6_, X represent any amino acid except cysteine; Supplementary Figures S1 and Figure 1) (Zhou, 2010). Moreover, PyasOBP2 shared the highest sequence identity (76.27%) with SoryGOBP83a and SzeaOBP8, followed by DponOBP3 (75.21% identity), DadjOBP6 (74.79% identity) and *Dendroctonus armandi* DarmOBP2 (73.50% identity). The phylogenetic tree showed that 40 coleopteran insect OBPs could be divided into three subfamilies: minus-C OBPs, classic OBPs and plus-C OBPs (Figure 2). Among the OBPs, the closest homolog of PyasOBP2 was SzeaOBP8, consistent with the results of multiple sequence alignments.

### Expression Profiles of *PyasOBP2*

**FIGURE 3:**
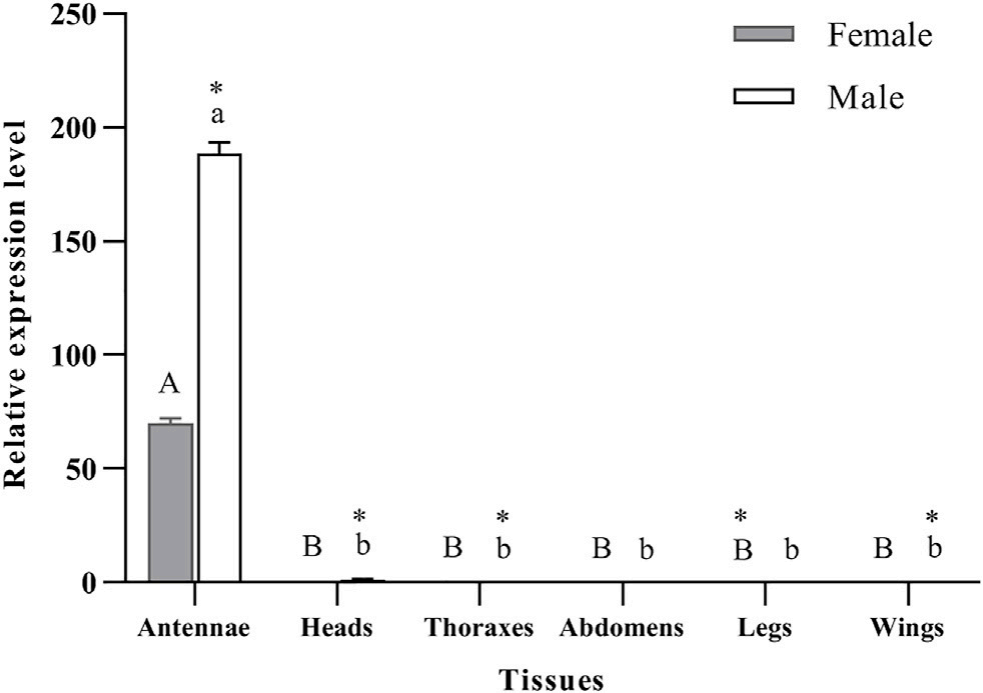
Expression profiles of the *PyasOBP2* gene in different tissues of *Pachyrhinus yasumatsui*. The fold changes are relative to the expression levels in the legs of adult females. Different capital and lowercase letters above bars indicate significant differences among tissues of females and males, respectively. Asterisks indicate significant differences in expression levels between female and male in the same tissues.

qRT-PCR was used to determine the expression levels of *PyasOBP2* in different adult tissues of both sexes of *P. yasumatsui*. For both female and male adults, *PyasOBP2* was significantly and highly expressed in antennae, but it was almost not expressed in all other tissues (Figure 3; ♀: *F*_5, 12_ = 3158.41, *p* < 0.001; ♂: *F*_5, 12_ = 4049.09, *p* < 0.001). Sex-biased expression of *PyasOBP2* was found in antennae, heads, thoraxes, legs, and wings. Expression levels of *PyasOBP2* in antennae (*t* = 67.49, *p* < 0.001), heads (*t* = 13.92, *p* < 0.01), thoraxes (*t* = 9.26, *p* < 0.05) and wings (*t* = 6.55, *p* < 0.05) were significantly higher in males than those in females. Whereas expression levels of *PyasOBP2* in legs were significantly higher in females than in males (*t* = 112.53, *p* < 0.001).

### Expression and Purification of PyasOBP2

**FIGURE 4:**
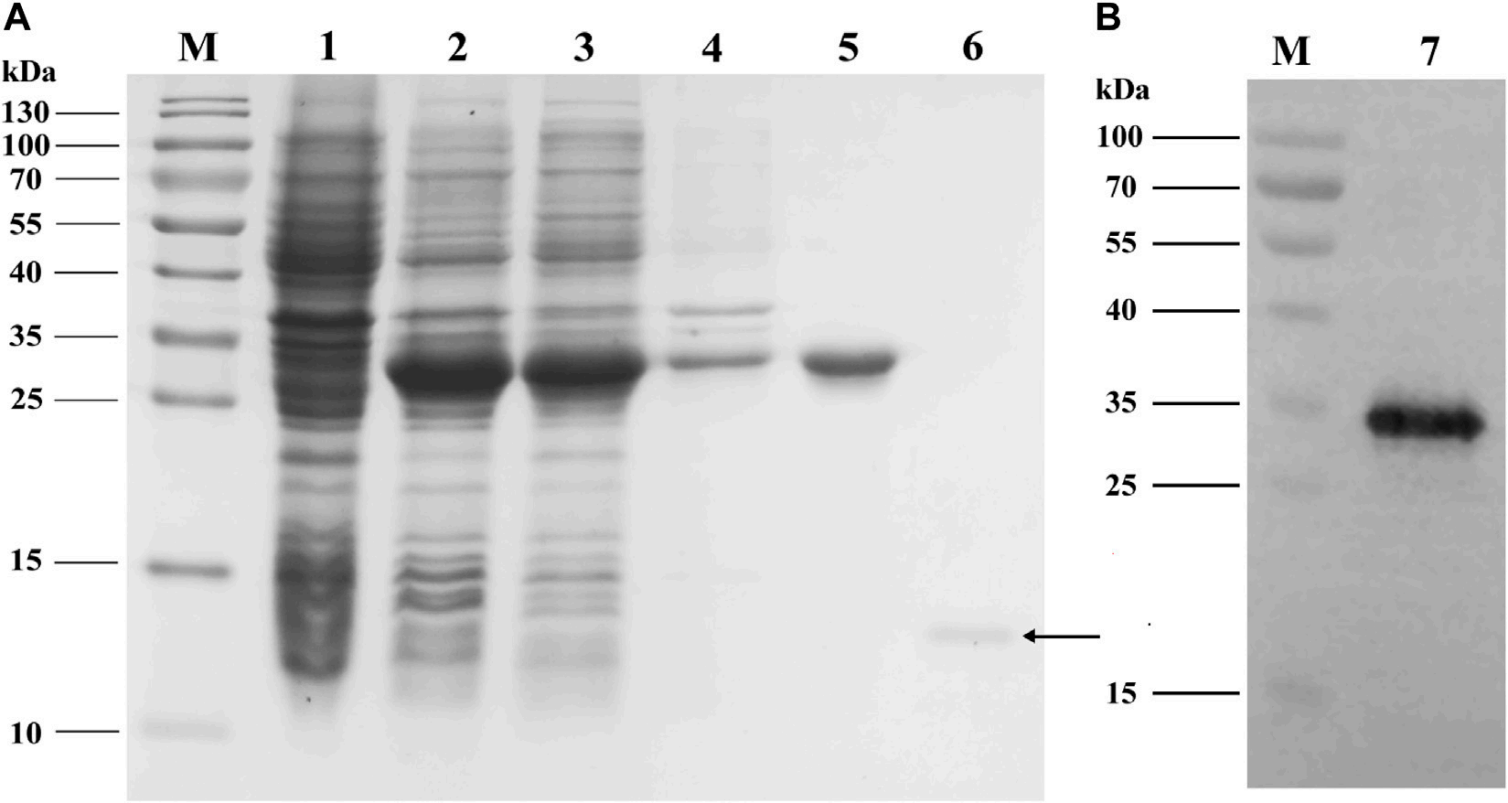
**(A)** SDS-PAGE and **(B)** western blot analyses of expressed recombinant PyasOBP2. M: Protein marker; 1: Non-induced pET32a/PyasOBP2; 2: Induced pET32a/PyasOBP2; 3: Supernatant of pET32a/PyasOBP2; 4: Inclusion body of pET32a/PyasOBP2; 5: Purified PyasOBP2; 6: Re-purified PyasOBP2 after the removal of His-tag; 7: Western blot of purified PyasOBP2.

The analyses of SDS-PAGE (Figure 4A) and western blot (Figure 4B) showed that recombinant PyasOBP2 was successfully expressed and purified with the *E. coli* system. The recombinant PyasOBP2 with His-tag was mainly present in the supernatant after IPTG induction, and exhibited distinct bands at the size of approximately 30 kDa. PyasOBP2 after the removal of His-tag had a high purity but low concentration (0.79 mg/ml), and showed a distinct band at the size of approximately 13.5 kDa (as shown by the arrow in Figure 4A).

### Fluorescent Competitive Binding Assays of PyasOBP2

**FIGURE 5:**
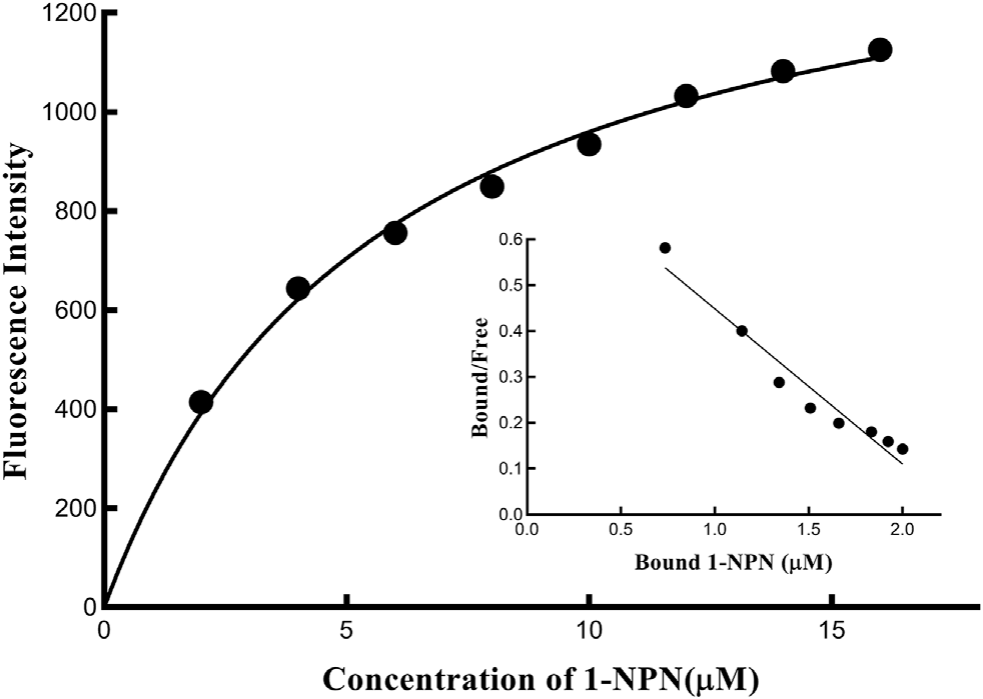
The binding curve and Scatchard plot of PyasOBP2 with 1-NPN.

**FIGURE 6:**
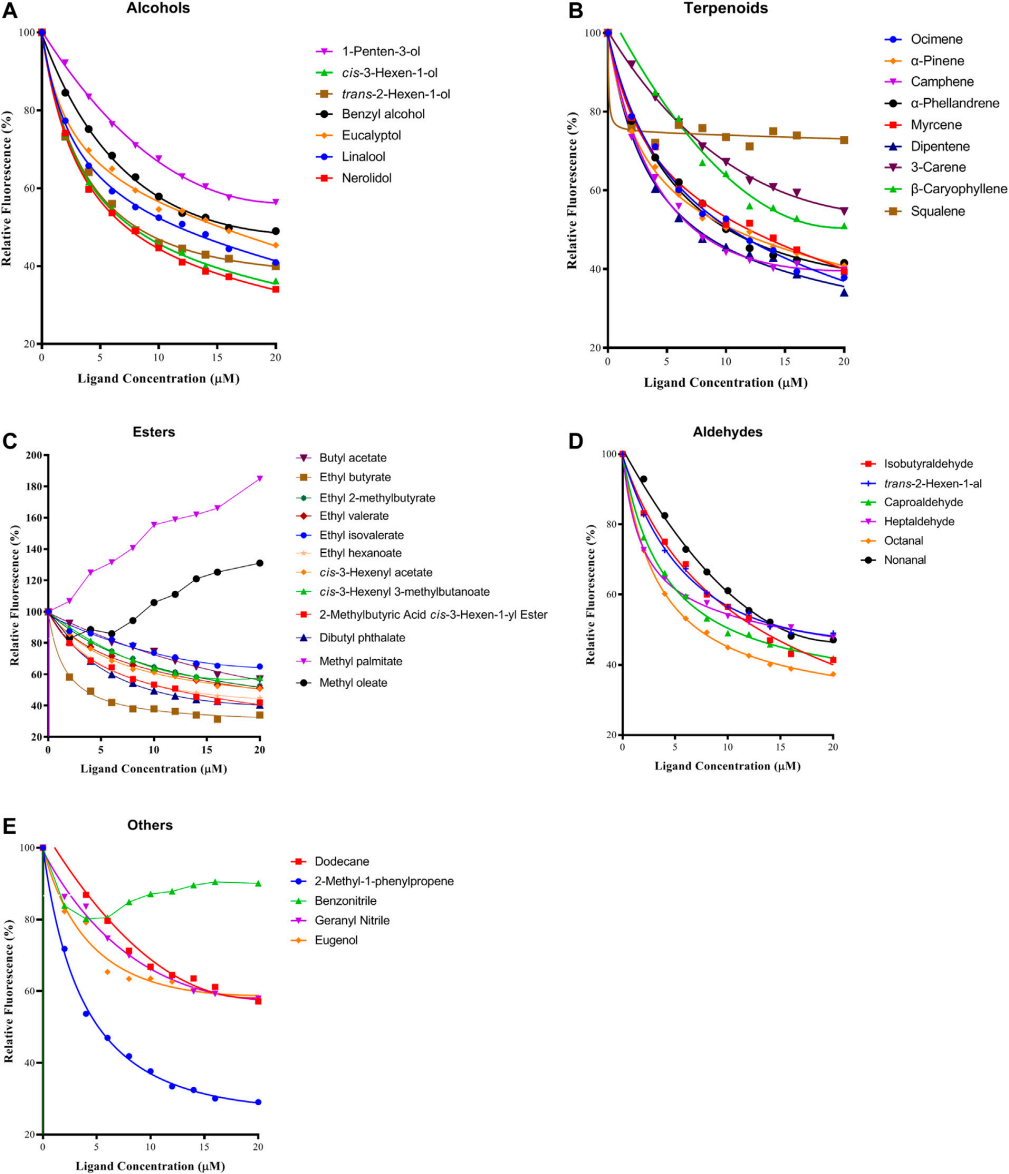
Binding curves of selected ligands to PyasOBP2. **(A)** Alcohols; **(B)** Terpenoids; **(C)** Esters; **(D)** Aldehydes; **(E)** Others.

The binding affinity of 1-NPN with the purified PyasOBP2 was measured, and the binding curve as well as corresponding Scatchard plot were drawn (Figure 5). Results revealed that the dissociation constant of PyasOBP2 with 1-NPN was 5.662 μM, suggesting 1-NPN is a good reporter ligand for PyasOBP2. Among 38 tested host volatiles, PyasOBP2 was found to bind to 22 volatiles (K_i_<20 μM), indicating that PyasOBP2 had a broad ligand-binding affinity (Table 2; Figure 6). Among the seven tested alcohols, cis-3-hexen-1-ol, trans-2-hexen-1-ol, linalool, and nerolidol showed strong binding affinity (K_i_<10 μM) for PyasOBP2 (Figure 6A). Among the nine tested terpenoids, six terpenoids (i.e., ocimene, α-pinene, camphene, α-phellandrene, myrcene, and dipentene) with the same molecular formula of C_10_H_16_, presented strong binding affinity for PyasOBP2 (K_i_ values = 5.99–9.18 μM) (Figure 6B). Among the eleven tested esters, only three esters, including ethyl butyrate, 2-methylbutyric acid cis-3-hexen-1-yl ester and dibutyl phthalate, displayed good binding affinity for PyasOBP2 (K_i_ values = 3.02–10.13 μM) (Figure 6C). All the six tested aldehydes showed good binding affinity for PyasOBP2 with K_i_ values ranging from 6.64 to 13.39 μM (Figure 6D). Among the five other ligands, 2-methyl-1-phenylpropene exhibited very strong binding affinity for PyasOBP2 (K_i_ = 4.61 μM), whereas dodecane, eugenol and two nitriles (benzonitrile and geranyl nitrile) could not bind to PyasOBP2 (Figure 6E).

### Protein Structure Prediction and Molecular Docking

**FIGURE 7:**
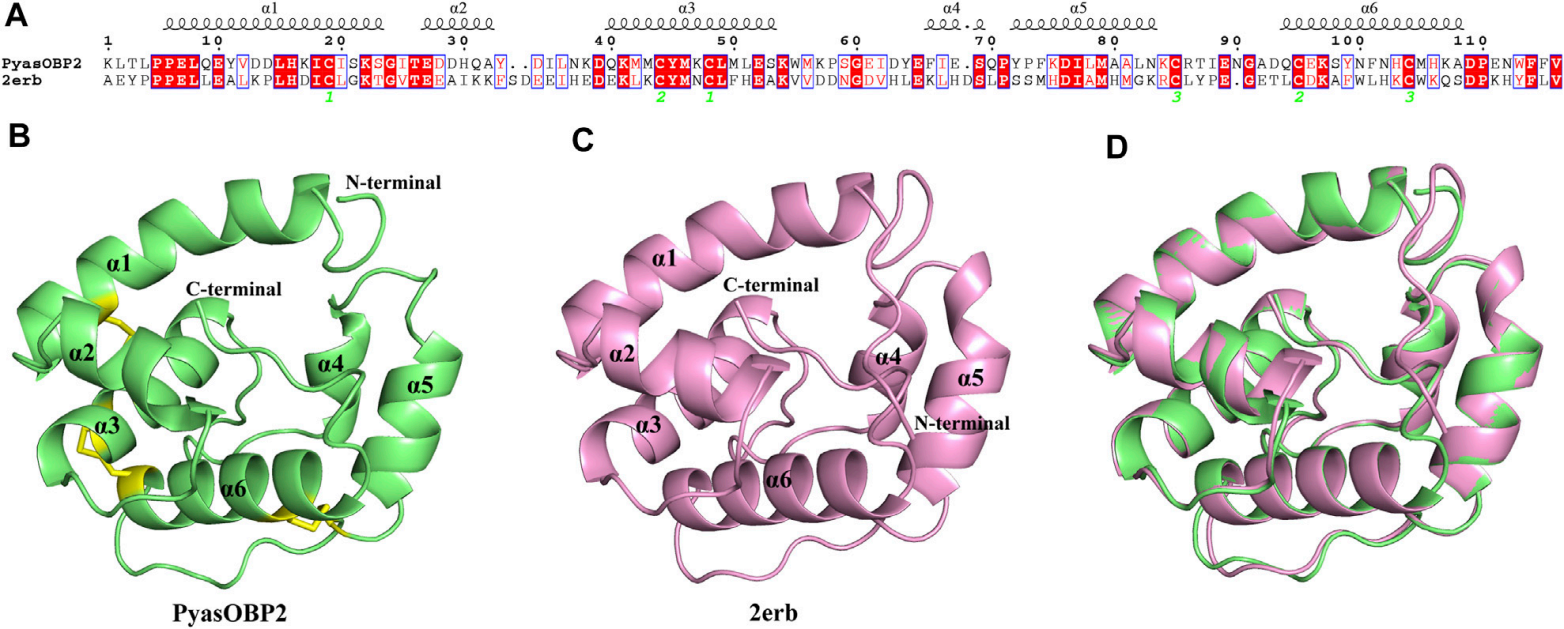
Structural modeling of PyasOBP2. **(A)** Sequence alignment between PyasOBP2 and AgamOBP1 (2erb). **(B)** 3D structure of PyasOBP2. The N-terminal, C-terminal and six α-helices are labeled, and three disulfide bridges are colored in yellow. **(C)** 3D structure of 2erb used as the template. **(D)** Superimposed structure of PyasOBP2 and the template 2erb.

BLAST results showed that PyasOBP2 shared the highest sequence similarity (34%) and query coverage (96%) with AgamOBP1. Therefore, *Anopheles gambiae* AgamOBP1 (2erb) was selected as the homology modeling template to generate 3D structure of PyasOBP2 (Figure 7). The obtained structural model of PyasOBP2 was evaluated by Verify3D and PROCHECK. In Verify3D analyses, 85.34% of residues had averaged 3D/1D score ≥ 0.2 (Supplementary Figure S2). The Ramachandran plot exhibited that 93.3% of amino acid residues were in most favored regions and only 1.0% of residues was in disallowed regions (Supplementary Figure S3), suggesting that the predicted model of PyasOBP2 is reasonable and reliable (Bowie et al., 1991; Lüthy et al., 1992). The predicted 3D structure of PysOBP2 consisted of six α-helices, including α1 (Pro6-Ser23), α2 (Glu27-Ala32), α3 (Gln40-Ser53), α4 (Phe66-Ser69), α5 (Tyr72-Asn83), and α6 (Gln94-Ala108) (Figure 7A). Among these, five α-helices (α1, α3, α4, α5 and α6), together with three pairs of disulfide bridges (Cys19 in α1 and Cys48 in α3, Cys44 in α3 and Cys95 in α6, Cys85 in α5 and Cys104 in α6), formed the hydrophobic binding pocket (Figure 7B).

**FIGURE 8:**
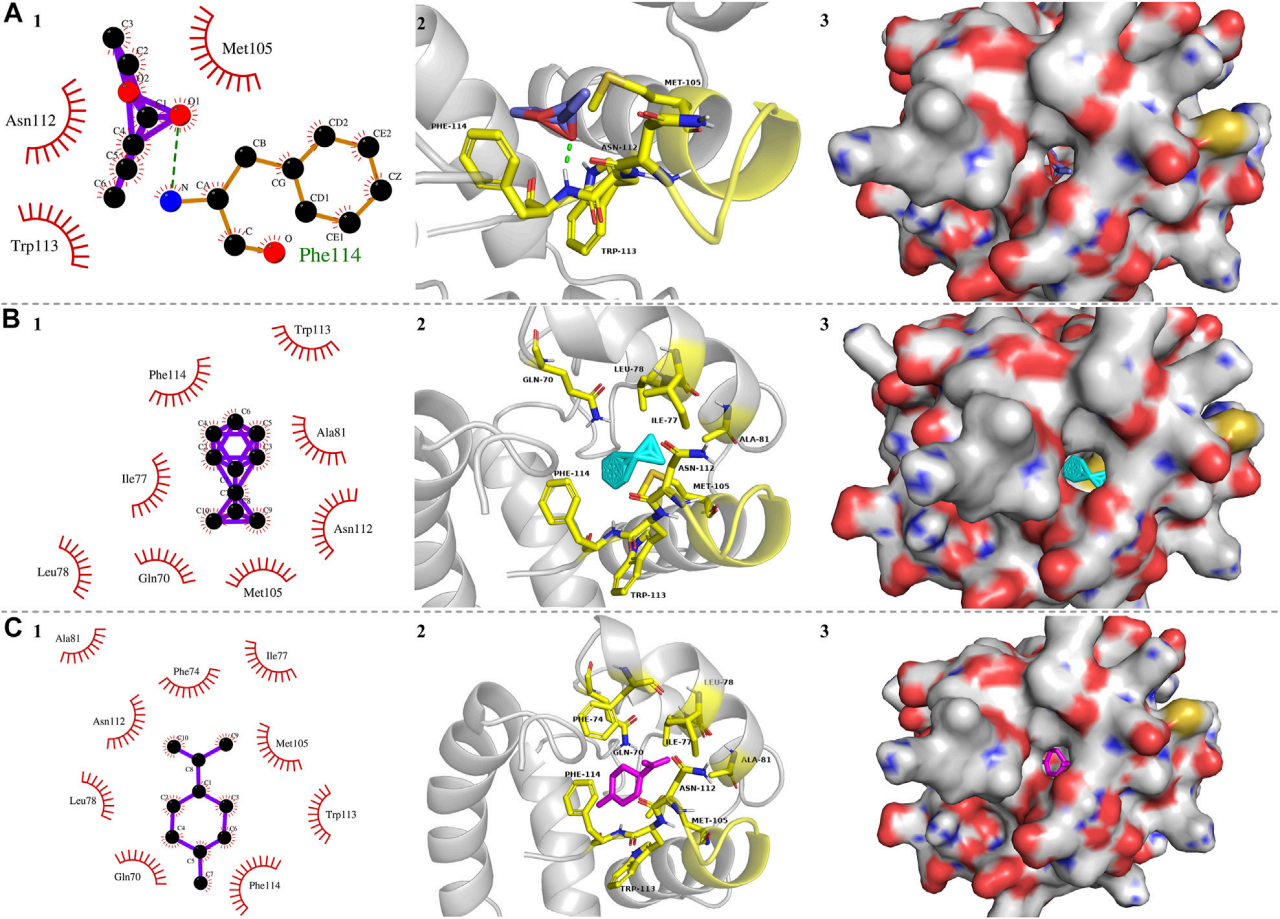
Molecular docking of PyasOBP2 with **(A)** Ethyl butyrate, **(B)** 2-Methyl-1-phenylpropene, and **(C)** Dipentene. **(1)** Key residues of PyasOBP2 involved in main interactions (2D). The hydrogen bond was marked with green dot line. **(2)** Key residues of PyasOBP2 involved in main interactions (3D). **(3)** The optimal orientation and conformation of different ligands in the hydrophobic cavity formed by hydrophobic residues of PyasOBP2.

To further explore the characteristics of PyasOBP2 binding sites, three ligands (i.e., ethyl butyrate, 2-methyl-1-phenylpropene, and dipentene), which exhibited very high binding affinities (K_i_ values from 3.02 to 5.99 μM) for PyasOBP2 in binding assays were selected for molecular docking. The docking results showed that the ligands bound in the PyasOBP2 pocket with negative energy values (Table 3). The 2D and 3D binding interactions, as well as the optimal orientation and conformation of three ligands in the hydrophobic cavity, were shown in Figure 8. We found that hydrogen bonds (Phe114), hydrophobic interactions (Met105) and van der Waals interactions (Asn112, Trp113) were the main interactions involved in binding of PyasOBP2 with ethyl butyrate (Table 3). For binding with 2-methyl-1-phenylpropene and dipentene, similar interactions were found involving main residues of Ile77, Leu78, Ala81, Met105, Phe114, Gln70, Asn112, and Trp113. Among these residues, Ile77, Leu78, Ala81, Met105, and Phe114 were mainly involved in hydrophobic interactions, whereas Gln70, Asn112, and Trp113 contributed the most to van der Waals interactions (Figure 8; Table 3).

**TABLE 3:** Docking results for PyasOBP2 with three ligands.

Ligandsact	Binding energy (Kcal/mol)	Residues involved in hydrogen bond	Residues involved in hydrophobic interactions	Residues involved in van der waals interactions
Ethyl butyrate	−3.71	Phe114	Met105	Asn112, Trp113
2-Methyl-1-phenylpropene	−5.85	-	Ile77, Leu78, Ala81, Met105, Phe114	Gln70, Asn112, Trp113
Dipentene	−5.92	-	Phe74, Ile77, Leu78, Ala81, Met105, Phe114	Gln70, Asn112, Trp113

## Discussion

In the present study, we cloned and characterized the OBP gene *PyasOBP2*, based on the antennal transcriptome of *P. yasumatsui*. PyasOBP2 has an N-terminal signal peptide containing 19 amino acids, and possesses six conserved cysteine residues that follow the typical pattern of classic OBPs: C_1_-X_24_-C_2_-X_3_-C_3_-X_36_-C_4_-X_9_-C_5_-X_8_-C_6_. Phylogenetic analyses showed that the closest homolog of PyasOBP2 was SzeaOBP8 from *S. zeamais* (76.27% sequence identity). Expression profile analyses showed that *PyasOBP2* was most highly expressed in the antennae of both males and females, but it was almost not expressed in all other tissues, implying that PyasOBP2 may play potential roles in perception of host plant odors (Sun et al., 2014; Li et al., 2017; He et al., 2019). Expression levels of *PyasOBP2* in antennae were significantly higher in males than those in females. This male-biased expression suggested that PyasOBP2 may detect pheromones released by females and play the same roles as pheromone binding proteins (PBPs) (Gong et al., 2014; Khuhro et al., 2017; Cui et al., 2018). However, sex pheromones are still unknown in *P. yasumatsui*. Thus, we leave this issue as a potential direction for future work.

OBPs were thought to be capable of binding to host plant volatiles (Vogt et al., 2015; Brito et al., 2016). In this study, we characterized the binding activities of PyasOBP2 to 38 selected volatiles from *Z. jujuba*. The fluorescence competitive binding assays showed that PyasOBP2 could bind with a wide range of volatile ligands (K_i_<20 μM), such as alcohols, terpenoids, esters and aldehydes, implying it had a broad ligand-binding affinity. Previous studies have proved that three plant volatiles, ocimene, nonanal and methyl palmitate, could elicite strong EAG responses in adult *P. yasumatsui* antennae (Yan et al., 2017; Yan et al., 2020). However, our results indicated that PyasOBP2 exhibited strong and moderate binding affinity with ocimene (K_i_<10 μM) and nonanal (K_i_<20 μM), respectively, whereas it was incapable of binding with methyl palmitate (K_i_>20 μM), suggesting that an OBP could only bind with some specific odors during the process of insect chemoreception (Zhang et al., 2020), and further studies on other OBPs from *P. yasumatsui* are needed to confirm this. Additionally, PyasOBP2 showed different binding affinities to some isomers, such as dipentene (K_i_<5 μM) and 3-carene (K_i_>20 μM), as well as ethyl butyrate (K_i_<5 μM) and butyl acetate (K_i_>20 μM), and it could not bind to some volatile ligands with more than 16 carbon atoms (such as methyl palmitate, methyl oleate and squalene), indicating that the size and structure of ligands, as well as their conformational changes, could affect the binding affinity for OBPs (Sandler et al., 2000; Christina et al., 2017).

In general, the 3D structure of OBPs contains a hydrophobic binding pocket formed by several α-helices, and some residues located in the pocket can be the potential binding sites in interactions between OBPs and ligands (Sandler et al., 2000). For instance, Tyr111 of HoblOBP1 is involved in the binding of hexyl benzoate (Zhuang et al., 2014); in LstiGOBP1, Thr15, Trp43, and Val14 play a key role in the binding wtih 1-heptanol (Yin et al., 2015), and Thr9, Val111, and Val114 are involved in the binding of dodecanol with GmolGOBP2 (Li et al., 2016). As the ligands with best binding affinity to PyasOBP2, ethyl butyrate, 2-methyl-1-phenylpropene, and dipentene were selected for docking with PyasOBP2. The molecular docking results showed that several hydrophobic residues (Leu8, Val12, Met46, Leu49, Met50, Trp55, Ile67, Gln70, Phe74, Ile77, Leu78, Ala81, Phe101, Asn102, and Met105) of PyasOBP2 could form a hydrophobic pocket important for ligand binding, and the residue Phe114 might contribute to the formation of hydrogen bonds in binding with some esters.

Except for hydrogen bonds, hydrophobic interactions and van der Waals interactions between insect OBPs and ligands are also crucial for ligand binding (Fu et al., 2018; Li et al., 2021). For binding with 2-methyl-1-phenylpropene and dipentene, Phe114 contributed the most to hydrophobic interactions. Met105 was mainly involved in hydrophobic interactions, whereas Asn112 and Trp113 might have affected the formation of van der Waals interactions in binding of PyasOBP2 with three ligands. Besides, the loop in the C-terminal of PyasOBP2 could act as a lid to cover the binding pocket, and some residues of this loop, such as Asn112, Trp113, and Phe114, could play a key role in the binding with some ligands. Similar results were reported in AgamOBP1 from *Anopheles gambiae* (Wogulis et al., 2006), HarmOBP7 from *Helicoverpa armigera* (Sun et al., 2013) and HoblOBP1 from *Holotrichia oblita* (Zhuang et al., 2014). Such a structure could function to prevent ligands from getting out of the pocket and strengthen the binding ability of PyasOBP2.

Overall, the OBP gene *PyasOBP2* from *P. yasumatsui* was reported for the first time, and this OBP demonstrated an antenna-specific expression pattern, as well as broad ligand-binding capability, providing evidence for the possible olfactory roles of OBPs in perceiving host plant odors of *P. yasumatsui*. Our molecular docking results indicated that the amino acid residue Phe114 of PyasOBP2 may be a key binding site, especially for some volatile ligands like ethyl butyrate, 2-methyl-1-phenylpropene and dipentene. In future studies, site-directed mutagenesis and RNAi experiments are needed to further clarify the importance of specific residues in PyasOBP2.

## Data Availability

The original contributions presented in the study are included in the article/Supplementary Material, further inquiries can be directed to the corresponding author.
