# Supplementary material for: Functional Characterization of Odorant Binding Protein PyasOBP2 From the Jujube Bud Weevil, Pachyrhinus yasumatsui (Coleoptera: Curculionidae)
Source: Front Physiol. 2022 Apr 27;13:900752. doi: 10.3389/fphys.2022.900752 (PMC9091336; doi:10.3389/fphys.2022.900752)
Supplement: Supplementary file 1 [file Table1.DOCX]

**Supplementary Materials**


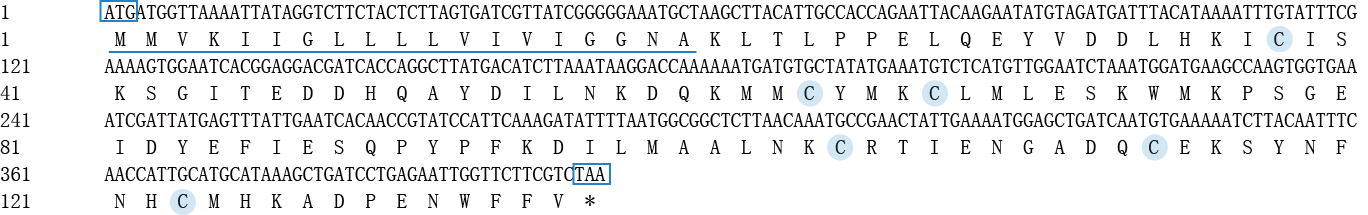


**Figure S1** Nucleotide and deduced amino acid sequences of PyasOBP2 in *Pachyrhinus yasumatsui.* Start and stop codons were boxed. The predicted signal peptide was underlined. The conserved cysteine residues were marked with a blue circle.


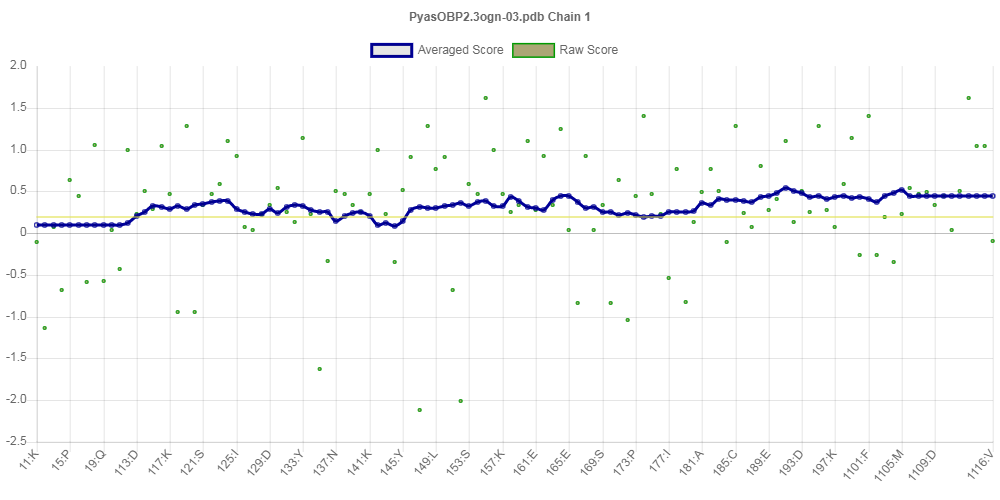


**Figure S2** Verify 3D results of the constructed 3D model of PyasOBP2


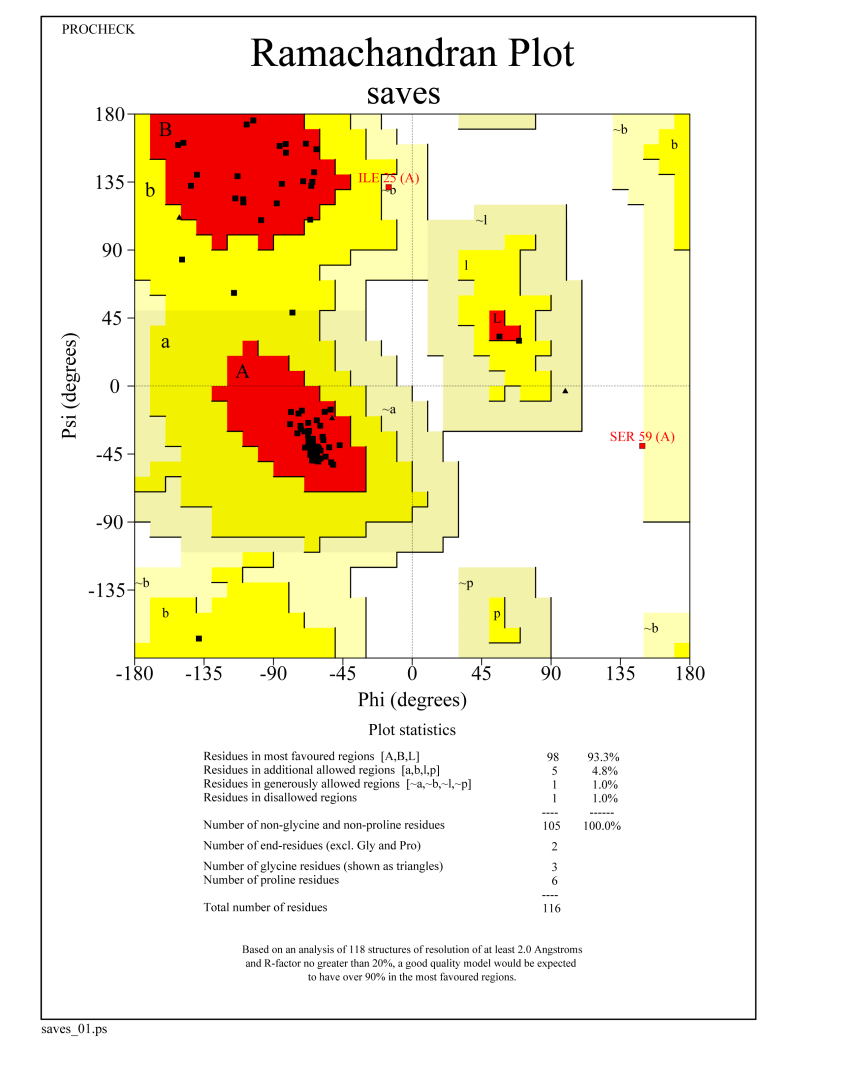


**Figure S3** Ramanchandran plot of the constructed 3D model of PyasOBP2
